# The FGFR2c/PKCε Axis Controls MCL-1-Mediated Invasion in Pancreatic Ductal Adenocarcinoma Cells: Perspectives for Innovative Target Therapies

**DOI:** 10.3390/biomedicines10071652

**Published:** 2022-07-09

**Authors:** Danilo Ranieri, Deborah French, Flavia Persechino, Luisa Guttieri, Maria Rosaria Torrisi, Francesca Belleudi

**Affiliations:** Department of Clinical and Molecular Medicine, Sapienza University of Rome, 00189 Roma, Italy; deborah.french@uniroma1.it (D.F.); flavia.persechino@uniroma1.it (F.P.); luisa.guttieri@uniroma1.it (L.G.); mara.torrisi@uniroma1.it (M.R.T.); francesca.belleudi@uniroma1.it (F.B.)

**Keywords:** Fibroblast Growth Factor Receptor 2 (FGFR2c), pancreatic ductal adenocarcinoma (PDAC), Protein-Kinase C Epsilon (PKCε), Myeloid Cell Leukemia 1 (MCL-1)

## Abstract

Pancreatic ductal adenocarcinoma (PDAC) is a lethal malignancy whose main characterizations are Kirsten Rat Sarcoma-activating mutations (KRAS) and a highly aggressive phenotype. Based on our recent findings demonstrating that the highly aberrant expression of the mesenchymal isoform of Fibroblast Growth Factor Receptor 2 (FGFR2c) in PDAC cells activates Protein-Kinase C Epsilon (PKCε), which in turn controls receptor-mediated epithelial to mesenchymal transition (EMT), here we investigated the involvement of these signaling events in the establishment of additional tumorigenic features. Using PDAC cell lines expressing divergent levels of the FGFR2c and stable protein depletion approaches by short hairpin RNA (shRNA), we found that FGFR2c expression and its PKCε downstream signaling are responsible for the invasive response to Fibroblast Growth Factor 2 (FGF2) and for anchorage-independent growth. In addition, in vitro clonogenic assays, coupled with the check of the amount of cleaved Poly Adenosine Diphosphate-Ribose Polymerase 1 (PARP1) by Western blot, highlighted the involvement of both FGFR2c and PKCε in cell viability. Finally, monitoring of Myeloid Cell Leukemia 1 (MCL-1) expression and Sarcoma kinase family (SRC) phosphorylation suggested that the FGFR2c/PKCε axis could control cell migration/invasion possibly via MCL-1/SRC-mediated reorganization of the actin cytoskeleton. Being PKCs RAS-independent substrates, the identification of PKCε as a hub molecule downstream FGFR2c at the crossroad of signaling networks governing the main malignant tumor hallmarks could represent an important advance towards innovative target therapies overcoming RAS.

## 1. Introduction

Pancreatic ductal adenocarcinoma (PDAC) is among the most lethal malignancies whose main characterization is the high frequency of Kirsten Rat Sarcoma-activating mutations (KRAS) [1,2,3].

In addition to KRAS, these cancers present a spread of mutations, involving several genes encoding for different signaling molecules, which makes it very difficult to determine clinical subtypes [1]. Moreover, the PDAC tumor microenvironment (TME), characterized by altered biochemical and biophysical conditions coupled with desmoplastic stroma and close crosstalk between cancerous and stroma cells, favors tumor progression and chemoresistance and leads to cancer immune evasion [1]. Even if the direct targeting of some of the involved signaling molecules and immune therapy, coupled with a variety of combinations of conventional drugs displayed the most promising results in pancreatic cancer treatment [1], they are still scarcely encouraging. In light of this and considering that KRAS is currently considered an “undruggable” molecule, identifying new pivotal signaling molecules (possibly acting bypassing KRAS) could help to overcome the obstacles in the way of treatment of one of the most stubborn cancers.

Protein Kinase C-mediated (PKC) signaling has been described as among the main RAS-independent pathways activated by several receptor tyrosine kinases (RTKs), including Fibroblast Growth Factor Receptors (FGFRs) [4], whose dysregulation significantly contributes to cancer development [5]. Concerning this topic, we have recently demonstrated a central contribution for the PKCε isoform in the induction of epithelial to mesenchymal transition (EMT) [6,7], a pathological event triggered by the mesenchymal isoform of Fibroblast Growth Factor Receptor 2 (FGFR2c), when aberrantly expressed in the epithelial contexts [6,7,8,9].

As mentioned above, the desmoplastic stroma is a typical feature of PDAC [10,11], in which the dysregulated signaling of several mitogenic growth factors, such as Epithelial Growth Factor (EGF), Insuline-Like Growth Factor (IGF), Platelet-Derived Growth Factor (PDGF) and Vascular-Endothelial Growth Factor (VEGF) appear to play important roles [1]. Among them, Fibroblast Growth Factor (FGF) is increasingly recognized as critical player in tumor/stroma cell crosstalk, pointing to the FGF/FGFR axis as a suitable target for combined therapies [12]. PDAC-derived tissues and cell lines display different patterns and amounts of the four FGFR family members, with FGFR1 and FGFR2 appearing the most involved in oncogenesis and FGFR4 playing an opposite tumor-suppressive function [12]. Interestingly, the involvement of FGFR1 has been specifically described in the MIAPaCa-2 cell line, which poorly expresses FGFR2 [13], while we recently highlighted a crucial involvement of the mesenchymal FGFR2c isoform in Pancreatic Adenocarcinoma 1 cells (PANC-1), which highly express this receptor [14]. FGFR1 and FGFR2c enhance alternative oncogenic pathways (Protein Kinase B (Akt)/Myelocytomastosis Oncogene (MYC) and PKCε/Extracellular Signaling-Regulated Kinase 1-2 (ERK1-2), respectively), both potentially bypassing RAS signaling, and resulting in different oncogenic outcomes (increased cell proliferation and enhanced EMT, coupled with dysregulated autophagy, respectively) [13,14]. These observations strongly suggest that the FGFR expression profile could significantly contribute to PDAC tumor cell plasticity, determining the response to paracrine FGFs and consequently the efficacy of the FGF/FGFR axis-based target therapies. However, in this scenario the involvement of the FGFR2c/PKCε signaling axis in cell survival and in the acquisition of an invasive phenotype, which is a crucial hallmark for tumor malignancy, is still not completely understood. For this reason, we have been motivated to focus our attention again on PDAC cells highly expressing FGFR2c, investigating the impact of this receptor and its downstream PKCε signaling on cell survival and invasion and searching for the downstream molecular events possibly involved.

Therefore, the rationale of this work has been to identify new signaling molecules whose targeting could be a promising perspective for combined therapies aimed to counteract malignant progression of those PDAC subgroups highly expressing FGFR2c.

## 2. Materials and Methods

### 2.1. Cells and Treatments

PANC-1 and the MIAPaCa-2 pancreatic adenocarcinoma cell lines were purchased from American Type Culture Collection (ATCC; Manassas, VA, USA) and cultured in Dulbecco’s modified Eagle’s medium (DMEM), supplemented with 10% fetal bovine serum (FBS) plus antibiotics. For FGFR2 and PKCε silencing, cells were stably transfected with Bek/FGFR2 Plasmid shRNA (Santa Cruz Biotechnology Inc., Santa Cruz, CA, USA, SC-29218-SH) and PKCε Plasmid shRNA (Santa Cruz Biotechnology Inc., Santa Cruz, CA, USA, SC-36251-SH) and shRNA as a control, using Lipofectamine 2000 transfection reagent (Life Technologies, Carlsbad, CA, USA; 11668-019) according to the manufacturer’s protocol. For growth factor stimulation, cells were left untreated or incubated with Fibroblast Growth Factor Receptor 2 (FGF2) (PeproTech, London, UK, BFGF 100-188) 25 ng/mL for 24 h at 37 °C. To induce activation and signaling of FGFR2 mesenchymal isoform, cells were serum starved and incubated with FGF2 (PeproTech, London, UK) 100 ng/mL for 10 min at 37 °C. For inhibition of FGFR2 tyrosine kinase activity, cells were pre-incubated with a specific FGFR2 tyrosine kinase inhibitor, 3-[4-Methyl-2-(2-oxo-1,2-dihydro-indol-3-ylidenemethyl)-1H-pyrrol-3-yl]-propionic acid (SU5402) 25 µmol/L (Calbiochem, Nottingham, UK, 572630) for 1 h before treatments with growth factors (GFs).

### 2.2. Western Blot Analysis

Cells were lysed in a buffer containing 50 mM 2-[4-(2-hydroxyethyl)piperazin-1-yl]ethanesulfonic acid (HEPES), pH 7.5, 150 mM Sodium Chloride (NaCl), 1% glycerol, 1% Triton X-100, 1.5 mM MgCl2, 5 mM ethylene glycol-bis(β-aminoethyl ether)-N,N,N′,N′-tetraacetic acid (EGTA), supplemented with protease inhibitors (10 g/mL aprotinin, 1 mM phenylmethylsulfonyl fluoride (PMSF), 10 µg/mL leupeptin) and phosphatase inhibitors (1 mM sodium orthovanadate, 20 mM sodium pyrophosphate, 0.5 M Sodium Fluoride (NaF)). A range of 20 to 80 µg of total protein was resolved under reducing conditions by 8 or 12% Sodium Dodecyl Sulphate - PolyAcrylamide Gel Electrophoresis (SDS-PAGE) and transferred to reinforced nitrocellulose (BA-S 83; Schleicher & Schuell, Keene, NH, USA, BA-S83). The membranes were blocked with 5% non-fat dry milk (Bio-Rad, Hercules, CA, USA, 170-6404) in Phosphate Buffered Saline (PBS) 0.1% Tween 20 (Bio-Rad, Hercules, CA, USA, 170-6531) and incubated with anti-Bek (C17) (Santa Cruz Biotechnology Inc., Santa Cruz, CA, USA, sc-122), anti-phospho- Fibroblast Growth Factor Receptor Substrate 2 α (FRS2-α) (Tyr196) (Cell Signaling Technology, Beverly, MA, USA, #3864), the anti-phospho- Sarcoma kinase (Src) Family (Tyr416, D49G4) (Cell Signaling Technology, Beverly, MA, USA #6943) monoclonal antibodies or anti-phospho-PKCε (Ser729) (Abcam, Cambridge, UK, ab63387) polyclonal antibodies followed by enhanced chemiluminescence (ECL) detection (Thermo Scientific, Rockford, IL, USA, 34580). The membranes were rehydrated by washing in PBS/Tween-20, stripped with 100 mM β-mercaptoethanol and 2% SDS for 30 min at 55 °C and probed again with anti-PKCε (Abcam Cambridge, UK; ab124806), anti-Src (Cell Signaling Technology, Beverly, MA, USA, #36D10), anti- Poly Adenosine Diphosphate-Ribose Polymerase 1 (PARP) (7D3-6) (BD Pharmingen, BD Bioscience, San Josè, CA, USA, 551024), anti- Myeloid Cell Leukemia 1 (Mcl-1) (D35A5) (Cell Signaling Technology, Beverly, MA, USA, #5453) monoclonal antibodies, anti-FRS2 (H-91) (Santa Cruz Biotechnology Inc., Santa Cruz, CA, USA, sc-8318), anti-α/β-Tubulin (Cell Signaling Technology, Beverly, MA, USA, #2148S) polyclonal antibodies or anti- Beta Actin (ACTB) (Sigma-Aldrich, Saint Louis, MO, USA, A5441) monoclonal antibodies to estimate the protein equal loading. Densitometric analysis was performed using Quantity One Program version 4.6.8 (Bio-Rad, Hercules, CA, USA). The resulting values from three different experiments (two for the anti-PARP1 blot) were normalized, expressed as fold increase respect to the control value and reported in graph as the mean values ± standard deviation (SD). Student’s t test was performed, and significance levels have been defined as *p* < 0.05.

### 2.3. Scratch Assay

Cells were seeded at 2.5 × 10^5^ cells on 35 mm plates and grown until confluence. Confluent cells were serum starved for 12 h and then a standardized cell-free area was introduced by scraping the monolayer with a sterile tip, as previously described [9].

Plates were photographed immediately after scratching, representing a T0 control (100% open area). After accurate wash, cells were incubated for 24 h with medium supplemented with 1% Fetal Bovine Serum (FBS) plus antibiotics in the presence or absence of FGF2. Phase contrast images were taken along the scratch at low magnification (10×) using an Axiovert 200 inverted microscope (Zeiss, Oberkochen, Germany). Cell migration was quantitated by measuring the gap distance between the scratch edges making three different measures for each photograph at top, center and bottom of the zoomed photo using the Axiovision software (Zeiss, Oberkochen, Germany). Gap distance was expressed as the mean percentage of residual open area compared to the respective cell-free surface in T0 (100%). The results are expressed as the mean values of three independent experiments ± SD. *p* values were calculated using Student’s t test and the significance level has been defined as *p* > 0.05.

### 2.4. Invasion Assay

A migration assay was performed using 24-well Transwell migration Boyden chambers (8 μm pore size; Costar, Cambridge, MA, USA) precoated with Matrigel (BD Biosciences, Bedford, MA, USA) diluted 1:2 in DMEM. 2.5 or 5 × 10^4^ cells were seeded in each filter and serum starved for 4 h at 37 °C. To induce chemotaxis: serum free medium, complete medium, FGF2 100 ng/mL in presence or not of SU5402 as above were added to the lower chamber. After 48 h, cells on the upper side of membranes were removed, while cells migrated on the bottom side were fixed in methanol and stained with toluidine blue. Quantitative analysis was assessed counting for each sample the migrated cells in 10 microscopic fields (magnification: 20×) from three independent experiments. Results have been expressed as the mean values ± SD. *p* values were calculated using Student’s t test and the significance level has been defined as *p* > 0.05.

### 2.5. Colony-Formation Assay

Cells were seeded in 6 well plates at a density of 10^3^ cells/well. The medium was renewed every three days for three weeks. Then, cells were fixed and stained with 0.1% crystal violet with anhydrous ethanol 10% for 15 min at room temperature then washed abundantly in PBS. Plates have been photographed and quantitative analysis was assessed counting the stained colonies from three independent experiments performed in triplicate. Results have been expressed as the mean values ± SD. *p* values were calculated using Student’s t test and the significance level has been defined as *p* > 0.05.

### 2.6. Soft Agar Colony Formation Assay

Cells were trypsinized and resuspended (5 × 10^3^ cells) in 3 mL of complete medium containing 0.4% agar. Cell suspension was added to 0.6% agar layer in six well plates. Immediately after plating, only single cells were visible in each well. Complete medium (0.5 mL) was added every three days and after three weeks, quantitative analysis was performed counting for each sample only >50 cells soft agar colonies from three independent experiments. Results have been expressed as the mean values ± SD. *p* values were calculated using Student’s t test and the significance level has been defined as *p* > 0.05.

## 3. Results

### 3.1. PKCε Phosphorylation/Activation Takes Place Downstream Highly Expressed FGFR2c

**Figure 1:**
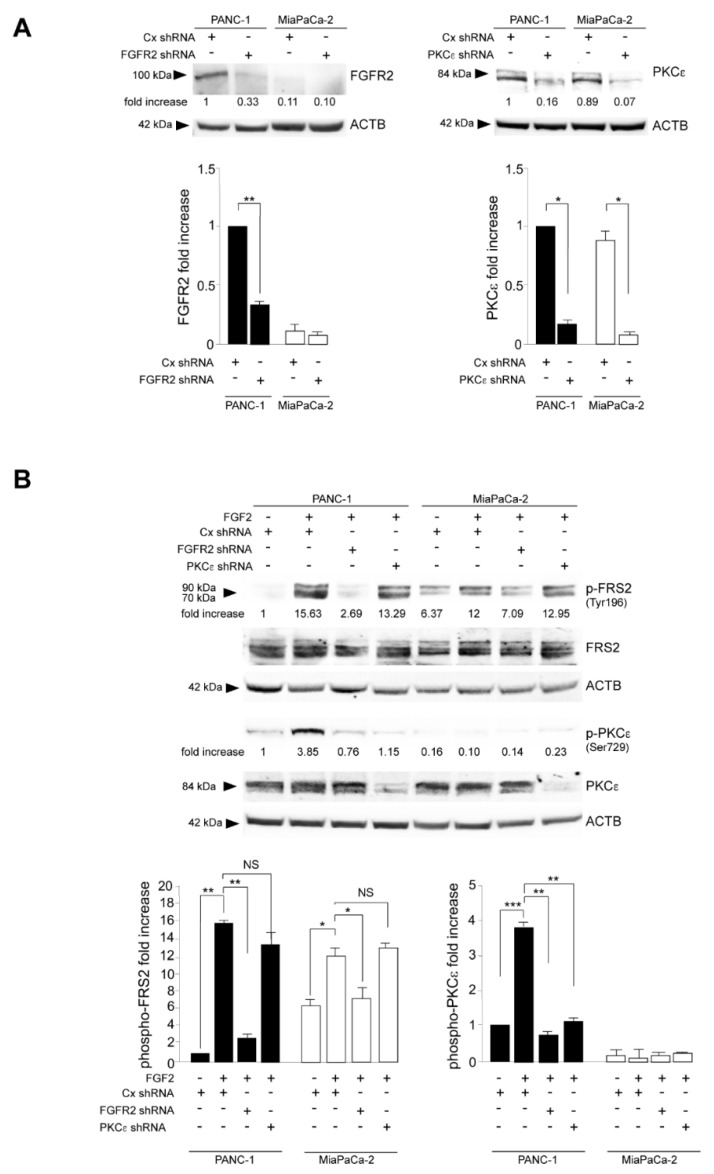
Protein-Kinase C Epsilon (PKCε) phosphorylation/activation takes place downstream highly expressed FGFR2c. Pancreatic adenocarcinoma 1 (PANC−1) and MIAPaCa-2 cells were stably transfected with Fibroblast Growth Factor Receptor (FGFR2) Short Hairpin RNA (shRNA) or with PKCε shRNA. Unrelated shRNA (Cx shRNA) was used as negative control. Cells were left unstimulated or stimulated with Fibroblast Growth Factor 2 (FGF2) for 10 min as reported in material and methods. (**A**) Western blot analysis shows the efficiency of the stable protein depletion of FGFR2 or PKCε by shRNA. (**B**) The enhancement of Fibroblast Growth Factor Receptor substrate 2 (FRS2) phosphorylation (Tyrosine 196 site) after FGF2 stimulation is higher in PANC−1 than in MIAPaCa-2 cells and is abolished by FGFR2 depletion, while remains unaltered after PKCε silencing. The phosphorylation of PKCε at Ser729 site in response to FGF2 is visible only in PANC-1 cells and is clearly dampened by either FGFR2 or PKCε depletion. Equal loading was assessed with the anti-actin antibody. For the densitometric analysis, the values from three independent experiments were normalized, expressed as fold increase and reported in graph as the mean values ± standard deviation (SD). Student’s t test was performed and significance levels have been defined as *p* < 0.05: *p < 0.05, ** *p* < 0.01, *** *p* < 0.001, NS: no significance.

To analyze the contribution of FGFR2c and of its downstream PKCε signaling in the establishment of late oncogenic features, such as cell invasion, in PDAC tumor cells, we first confirmed the relationship between FGF2-mediate activation of FGFR2c and PKCε signaling and their dependence on FGF2c expression levels. To this aim, stable protein depletion of FGFR2 or PKCε by short hairpin RNA (shRNA) was performed in PANC-1 and MIAPaCa-2: two PDAC cell lines expressing opposite levels of the mesenchymal FGFR2c isoform, and very poor levels of the epithelial FGFR2b variant [14,15,16]. Previous experiments performed by us using both FGFR2 shRNA- and FGFR2 isoform-specific FGFR2b and FGFR2c small interfering RNAs (siRNA), have assessed that, especially in PANC-1 cells, the generic FGFR2 shRNA efficiently caused a specific depletion of the FGFR2c variant [14]. The efficiency of the gene silencing performed by FGFR2 and PKCε shRNAs was assessed by Western blot analysis (Figure 1A), which also confirmed the evident divergence of the two cell lines in terms of FGFR2c expression (Figure 1A, left panel). Then, cells were stimulated with FGF2 and the extent of FGFR2c activation was assessed monitoring the phosphorylation of FRS2, a signaling platform constitutively linked to FGFR2 and widely recognized as molecular sensor of FGFR2 activation [17]. PKCε activation was assayed in parallel analyzing the phosphorylation of Serine 729 site, which is a widely recognized indicator of PKCε activation [18,19] Western blot analysis showed a FRS2 phosphorylation in response to FGF2 stimulation, more intense in PANC-1 cells (Figure 1B), which was abolished by FGFR2 depletion, but remained unaltered after PKCε silencing (Figure 1B). In contrast, the phosphorylation of PKCε in response to FGF2 was detectable exclusively in PANC-1 cells (Figure 1B) and was clearly dampened by either FGFR2 or PKCε depletion (Figure 1B), confirming that PKCε signaling take place downstream FGFR2c and it appears to depend on the receptor expression levels.

### 3.2. The Invasive Response to FGF2 can Be Ascribed to FGFR2c-Mediated PKCε Signaling

**Figure 2:**
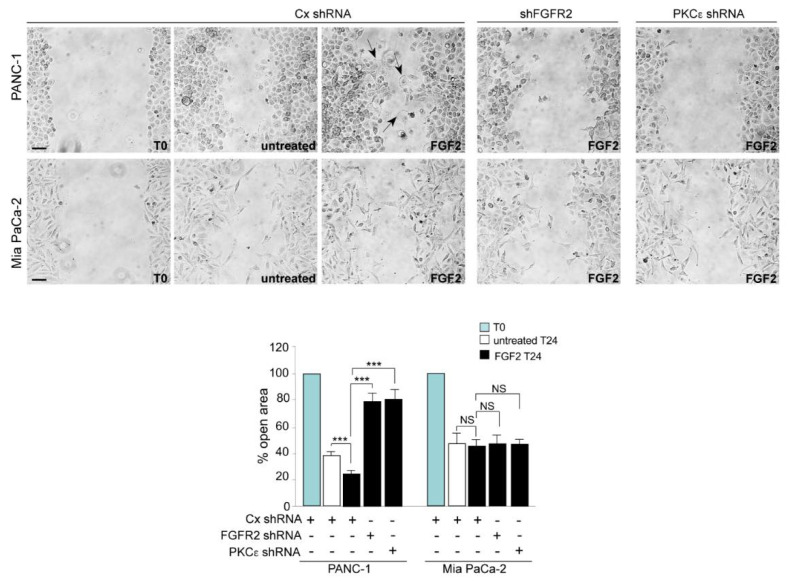
PANC-1 cell migration in response to FGF2 is impaired by FGFR2c or PKCε depletion. PANC−1 and MIAPaCa-2 cells were transfected as described above and then grown until confluence. A cell-free area was introduced in the monolayer using a tip. Cells were then immediately photographed (T0) or cultured in the presence or not of FGF2 for 24 h (T24). The in vitro scratch assay shows a specific migratory response to FGF2 only in PANC-1 cells. Lamellipodia and ruffles are evident at the leading edge of PANC-1cells distributed in the scratch area (arrows). The repopulation of the scratch area in PANC-1 cultures is impaired by FGFR2 and/or PKCε depletion. Quantitative analysis of cell migration was assessed measuring the gap distance between the edges of the scratch area as reported in materials and methods. Results are expressed as the mean value of three independent experiments ± (SD). Student’s t test was performed and significance levels have been defined as *p* < 0.05: *** *p* < 0.001, NS: no significance. Bar: 50 µm.

We recently found that the high expression of FGFR2c in PDAC cells efficiently activates PKCε signaling, which results in an enhancement the Epithelial Mesenchymal Transition (EMT) program [14]. Since we previously observed that, in normal human keratinocytes, FGFR2c-mediated EMT and cell migration possibly synergized contributing to cell invasion [9,20], we checked if, also in the PDAC context, FGFR2c expression and its downstream PKCε signaling could impact on cell motility. The in vitro scratch assay showed that a specific migratory response to FGF2 was detectable only in PANC-1 cells (Figure 2). The presence of motile structures, such as lamellipodia and ruffles, at the leading edge of cells distributed in the scratch area (Figure 2, arrows) further confirmed the mitogenic effect. Interestingly, only in PANC-1 cells was the repopulation of the scratch area impaired by FGFR2 and/or PKCε depletion (Figure 2), confirming its close dependence on high receptor expression and PKCε signaling.

**Figure 3:**
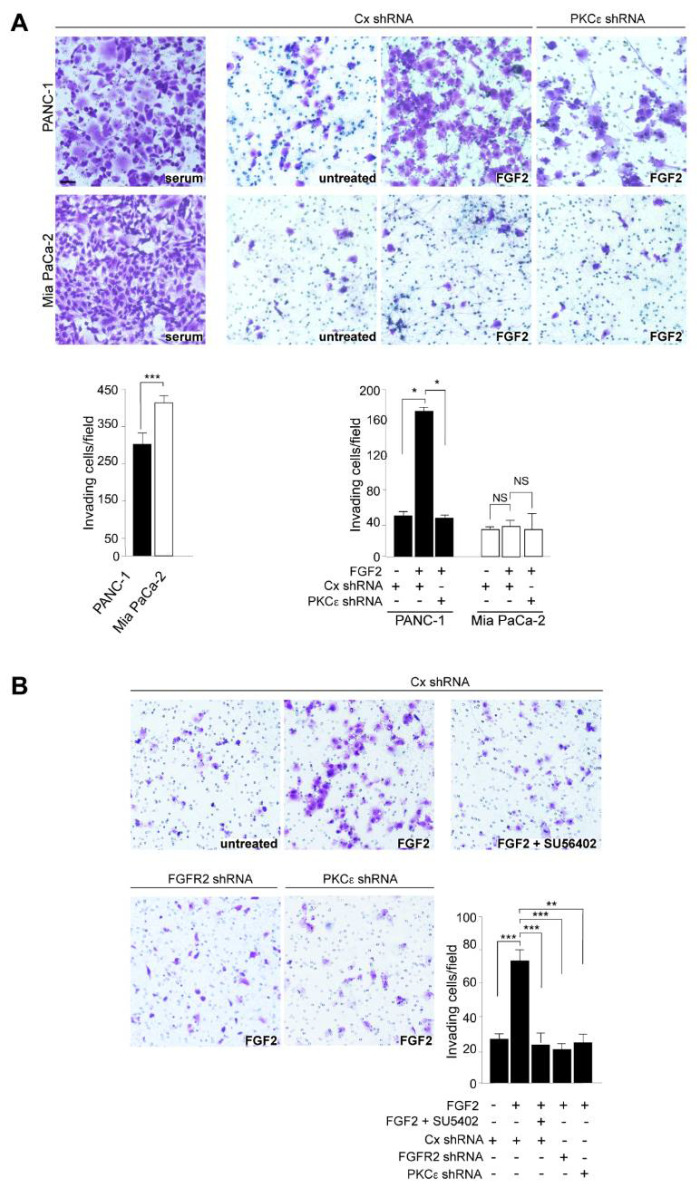
FGF2-induced invasive behavior of PANC-1 cells is counteracted by the interference with FGFR2c/PKCε signaling. PANC−1 and MIAPaCa-2 cells were transfected as described above and then seeded on Matrigel pre-coated Transwell Boyden chamber filters. Cells were then serum starved and complete medium or medium containing FGF2, in presence or not of SU5402, was added in the bottom chamber for 48 h, to stimulate cell chemotaxis. (**A**) MIAPaCa-2 cells show a higher invasive ability in complete medium, but only PANC-1 cells display an evident FGF2-specific invasive response, which is significantly dampened by PKCε depletion. (**B**) PANC-1 cells were transfected, seeded on Matrigel pre-coated chambers and stimulated with FGF2 as reported above: FGF2-induced invasion is inhibited by 3-[4-Methyl-2-(2-oxo-1,2-dihydro-indol-3-ylidenemethyl)-1H-pyrrol-3-yl]-propionic acid (SU5402) or FGFR2 silencing, as well as by PKCε depletion. Quantitative analysis was assessed as reported in materials and methods. Results are expressed as the mean value of three independent experiments ± (SD). Student’s t test was performed and the significance level has been defined as *p* < 0.05: * *p* < 0.05, ** *p* < 0.01, *** *p* < 0.001, NS: no significance. Bar: 20 µm.

Based on our previous findings highlighting the role of FGFR2c and its downstream PKCε signaling in conferring invasiveness to non-invasive normal keratinocytes [6,9,20], in a second step we assessed the impact of the FGFR2/PKCε axis on the widely described ability of PANC-1 and MIAPaCa-2 cells to migrate through Transwell Boyden chambers pre-coated with a thin layer of Matrigel, mimicking the in vivo basement membrane [21]. Upon seeding, cells were left in serum-enriched medium, or serum-starved overnight and then stimulated with FGF2 for 48 h. The results showed that, even if MIAPaCa-2 cells showed a higher invasive ability, when grown in complete medium (Figure 3A), only PANC-1 cells displayed an evident FGF2-specific invasive response, which was significantly dampened by PKCε silencing (Figure 3A). A comparable repression of FGF2-dependent invasion in these cells was observed after FGFR2 signaling shut-off by the FGFR tyrosine kinase inhibitor SU5402 or FGFR2 silencing (Figure 3B), confirming the dependence of PKCε signaling on FGFR2 expression and activation.

**Figure 4:**
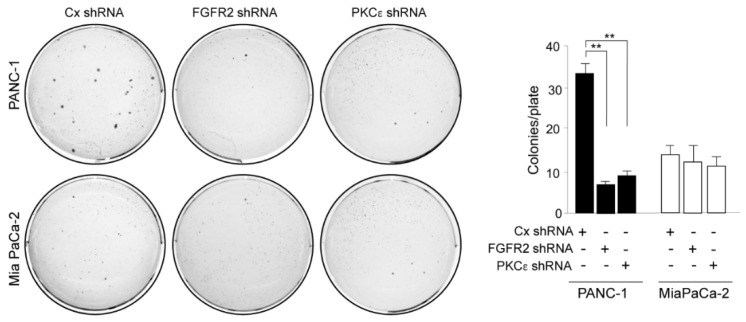
Anchorage-independent growth of PANC-1 cells is dampened by FGFR2c or PKCε depletion. PANC−1 and MIAPaCa-2 cells were transfected as described above and then trypsinized and resuspended in complete medium containing agar. Only in PANC-1 cells does the depletion of FGFR2 or PKCε negatively impact on the number of colonies formed after 3 weeks. Results are expressed as the mean value of three independent experiments ± (SD). Student’s t test was performed and significance levels have been defined as *p* < 0.05: ** *p* < 0.01.

The evidence of a contribution of FGFR2c expression and signaling in the establishment of an invasive response to FGF2, encouraged us to also investigate their possible involvement in the modulation of other malignant tumorigenic features, such as the anchorage-independent growth. To check this ability, the soft agar assay has been chosen, because it represents a reliable surrogate assay for estimating in vivo tumorigenesis. Results revealed that, differently from what observed in MIAPaCa-2 cells (Figure 4), in PANC-1 cells, the depletion of FGFR2 or PKCε negatively impacted on the number of suspended colonies formed in 3 weeks (Figure 4).

Thus, the expression rate of FGFR2c and the consequent establishment of a sustained PKCε signaling appear to significantly impact on PANC-1 cell behavior in terms of invasiveness in response to paracrine factors and anchorage-independent growth: two crucial in vitro parameters to predict their in vivo malignant potential.

### 3.3. FGFR2c/ PKCε Signaling Controls the Positive Interplay between Cell Survival and Invasion through the Induction of MCL-1

**Figure 5:**
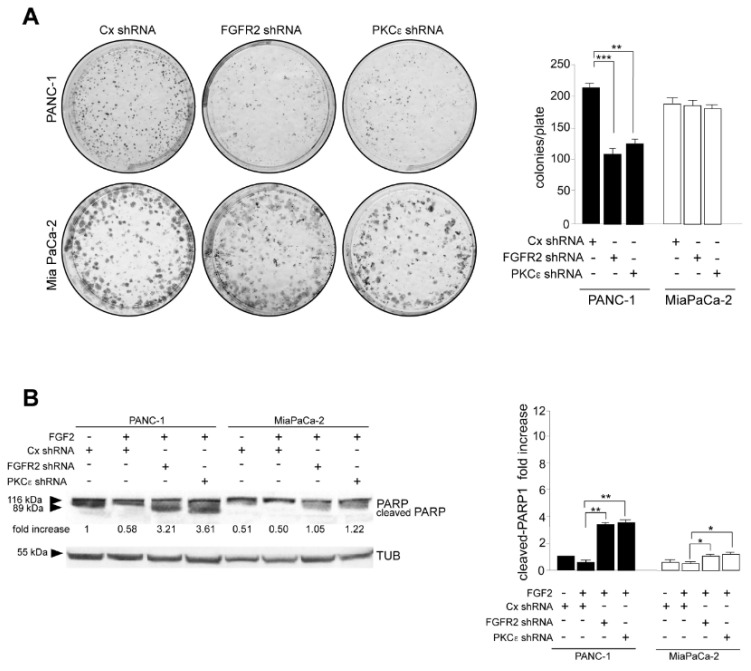
PDAC cell survival is counteracted by the repression of the FGFR2c/PKCε axis. (**A**) PANC−1 and MIAPaCa-2 cells were transfected, trypsinized and resuspended in complete medium as described above and finally seeded in 6 well plates. The depletion of both FGFR2 and PKCε drastically impacts on clonogenicity of PANC-1 cultures while no effects are appreciable in MIAPaCa-2 cells. Results are expressed as the mean value of three independent experiments ± (SD). Student’s t test was performed and significance levels have been defined as *p* < 0.05: ** *p* < 0.01, *** *p* < 0.001. (**B**) PANC−1 and MIAPaCa-2 cells were transfected and treated or not with FGF2 for 24 h as described materials and methods. Western blot show the appearance of the band corresponding to cleaved-PARP1 in consequence of FGFR2 or PKCε silencing, especially in PANC-1 cells. Equal loading was assessed with the anti-tubulin antibody. For the densitometric analysis, the values from three independent experiments were normalized, expressed as fold increase and reported in graph as the mean values ± (SD). Student’s t test was performed, and significance levels have been defined as *p* < 0.05: * *p* < 0.05, ** *p* < 0.01.

**Figure 6:**
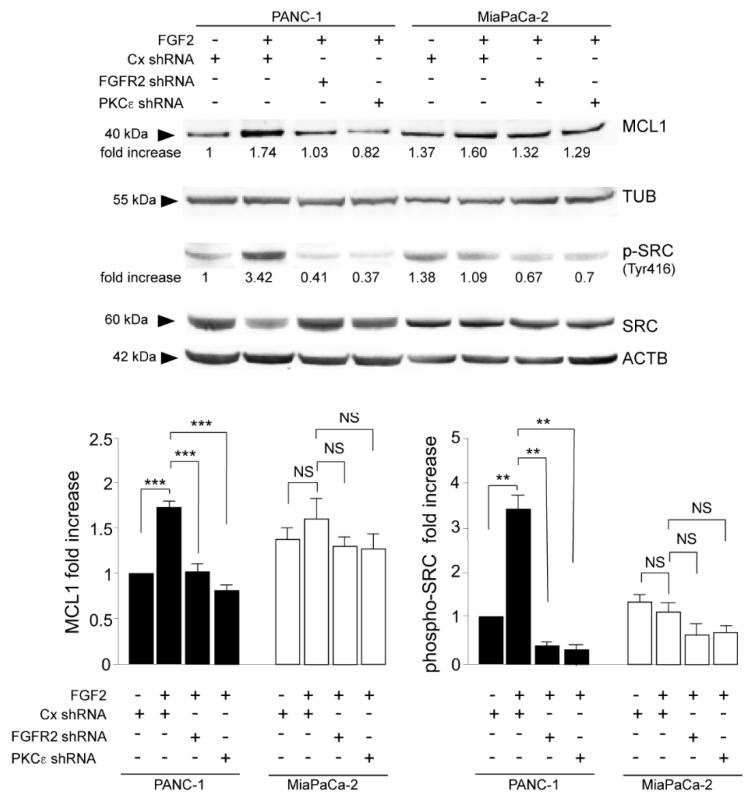
FGF2-mediated activation of FGFR2c/PKCε signaling increases Myeloid Cell Leukemia 1 (MCL-1) expression and and Sarcoma kinase family (SRC) phosphorylation/activation. PANC−1 and MIAPaCa-2-transfected cells were treated with FGF2 for 24 h as above. Western blot analysis shows that MCL-1 levels are increased by FGF2 stimulation, but significantly downregulated by FGFR2 or PKCε depletion in PANC-1 cells, while they remain almost unaltered in MIAPaCa-2 cells. A similar trend is observed for SRC phosphorylation (tyr416 site). Equal loading was assessed with the anti-tubulin or anti-actin antibody. For the densitometric analysis, the values from three independent experiments were normalized, expressed as fold increase and reported in graph as the mean values ± SD. Student’s t test was performed and significance levels have been defined as *p* < 0.05: ** *p* < 0.01, *** *p* < 0.001, NS: no significance.

In a next step, we assess the contribution of the FGFR2c/PKCε axis in determining PDAC cell viability, performing the in vitro colony formation assay. The results showed that the depletion of both FGFR2 and PKCε drastically impacted on clonogenicity of PANC-1 cultures (Figure 5A), while their effects on MIAPaCa-2 cells appeared almost negligibles (Figure 5A). Cell survival was further assessed by analyzing the levels of poly(ADP-ribose) polymerase 1 (PARP-1), which is among the cellular substrates of caspases [22]. Western blot analysis revealed that an appreciable appearance of the band corresponding to cleaved-PARP1 in consequence of FGFR2c or PKCε silencing was especially evident in PANC-1 cells (Figure 5B), confirming the anti-apoptotic effects of the FGFR2c/PKCε axis in these cells. Since cell survival and invasion are interplaying processes in cancer, our results suggest the idea that, in PDAC context, this interplay could be upstream regulated by FGFR2c. To assess it and to further clarify the molecular mechanisms possibly involved, we pointed our attention on MCL-1: the anti-apoptotic member of the B-cell lymphoma 2 family (BCL-2), whose protein levels correlate with malignancy, and drug resistance in many tumors, including pancreatic cancers [23,24,25,26,27,28]. MCL-1 can be induced by several signaling events, including FGFR activation [29], and it has been recently described as a hub molecule at the crossroad between cell survival and invasion, able to contribute to the actin cytoskeleton remodeling via SRC in several cancers [30], including triple-negative breast cancer [30] and PDAC [31]. Therefore, we checked the possible impact of the FGFR2c/ PKCε axis on MCL-1 activity in our PDAC cell models. Western blot analysis highlighted that MCL-1 levels were increased by FGF2 stimulation, but significantly downregulated by FGFR2c or PKCε depletion in PANC-1 cells (Figure 6), while they remained almost unaltered in MIAPaCa-2 cells (Figure 6). A similar trend was observed for SRC phosphorylation at the tyrosine 416, (Figure 6) which is an autophosphorylation site usually monitored to assess SRC kinase activation [32]. These results revealed the possible molecular cascade taking place downstream FGFR2c: only when highly expressed, FGFR2c triggers PKCε signaling, whose activation increases MCL-1 expression and consequently SRC activity, resulting in the enhancement of cell invasion.

## 4. Discussion

PDAC is an aggressive carcinoma whose KRAS constitutive activation is the main hallmark for malignancy [1,2,3]. However, accumulating observations suggested that, in specific contexts, KRAS could be dispensable [13,33,34], making its potentially overcoming its “undruggability”. Considering this possibility, research efforts have been recently focused on the identification of new signaling pathways, alternative to RAS, whose inhibition could be a promising strategy to be introduced in new therapies based on the combination of traditional chemotherapy, targeted therapies and immunotherapy. The abundance of these signaling pathways and the urgent need to dissect them for new treatment approaches has been recently highlighted in a recent review from Javadrashid and co-workers [1]. Among the described pathways, the FGF/FGFR axis has been identified as suitable therapeutic target thanks to its important contribution in regulating tumor/stroma cell crosstalk, particularly in PDAC [1,12].

Since the specific oncogenic contribution of FGFR2 isoform switch in PDAC remained for longtime controversial [15,16,35], we recently took advantage of the use of two cell lines expressing divergent levels of the mesenchymal FGFR2c isoform, demonstrating that the expression rate of this receptor is crucial in determining cell sensitivity to FGF2 in terms of EMT signature and autophagy repression [14]. We also found that PKCε is the downstream signaling molecule, activated only when FGFR2c is highly expressed, which is simultaneously involved in EMT enhancement and autophagy inhibition through a direct convergence on ERK1/2 pathway [14]. Therefore, supported by our previous data, in this work we further assessed the role of FGFR2c and its downstream PKCε signaling in PDAC malignant progression, analyzing their possible involvement in the establishment of further key tumorigenic features. Taking advantage of the use of the same cellular models previously used by us and using stable protein depletion approaches by shRNA, we confirmed that, when highly expressed, FGFR2c establish a FGFR2c/PKCε axis which is responsible for a FGF2-selective invasive response and for anchorage-independent growth. Moreover, colony formation assays and biochemical approaches aimed to check PARP1/cleaved PARP1 ratio, revealed that both FGFR2c and PKCε depletion significantly decreased cell viability. These results encouraged us to suppose that the interplay between cell survival and invasion could possibly upstream regulated by the highly expressed FGFR2c.

**Figure 7:**
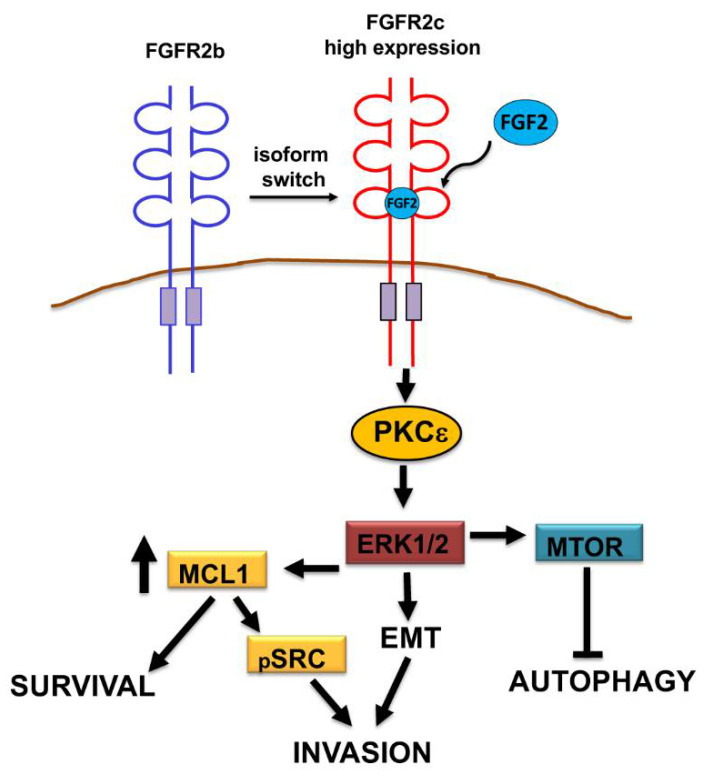
Schematic drawing of FGR2c/PKCε signaling network and its tumorigenic outcomes in PDAC cells. Only when highly expressed and in response to paracrine FGF2, FGFR2c triggers the activation of the FGFR2c/PKCε axis, which simultaneously controls, possibly converging on Extracellular Signaling-Regulated Kinase 1-2 (ERK1/2) pathway, EMT and repression of autophagy, as well as MCL-1-dependent cell survival and invasion.

We therefore attempt to identify the molecular mechanisms governing this interplay, focusing our attention on MCL-1: the BCL-2 family anti-apoptotic member recently identified as involved in the control of cell invasion in several tumors, including breast cancer [30] and PDAC [31]. Biochemical approaches demonstrated that, only in PANC-1 cells MCL-1 levels were increased in response to FGF2, but strongly decreased by FGFR2c or PKCε depletion and a similar trend was observed for the phosphorylation/activation of SRC. Since MCL-1 regulates cell invasion through SRC-mediated reorganization of the actin cytoskeleton [28,30,31], our results indicated that these molecular events are upstream controlled by the FGFR2c/PKCε axis. Our hypothesis is further sustained by a study, demonstrating that PKCη (another PKC family member belonging to the same isoform subgroup of PKCε) can regulate MCL-1 expression via ERK signaling [36]: the same downstream pathway to which converges the FGFR2c/PKCε axis in PDAC. Therefore, based on our and other observations, we can speculate that, only when highly expressed and in response to paracrine factors, FGFR2c triggers the activation of the FGFR2c/PKCε axis, which simultaneously controls several oncogenic events possibly converging on ERK1/2 pathway, including EMT and repression of autophagy [14], but also MCL-1-dependent cell survival and invasion (Figure 7). It is well known that pancreatic cancer is characterized by mutations in a high number of genes codifying for wide variety of signaling molecules, which justify the abondance of PDAC tumor cell phenotypes [1]. In light of this, we can speculate that the phenotypical traits observed by us for PANC-1 cells could be shared by those molecular subtypes highly expressing FGFR2c.

Moreover, being PKCs widely recognized as RAS-independent substrates, the identification of PKCε as a hub molecule at the crossroad of signaling networks governing the main tumor malignant hallmarks in PDAC could represent an important advance towards innovative target therapeutic strategies overcoming RAS.

Moreover, as mentioned above, PDAC tumor cells express different patterns of FGFRs. Importantly, different FGFRs display differential and sometime opposite (oncogenic vs. tumors-suppressive) functions in these cells [12], which could possibly underly the only partial efficacy of FGFR-target therapies in PDAC, substantially using pan-FGFR inhibitors. Our findings point to PKCε as a FGFR2c-specific signaling substrate in epithelial cells, which we have demonstrated not to be activated by other FGFRs physiologically expressed by normal epithelial contexts (such as FGFR2b) [6], often exerting tumor-suppressive roles in cancer. Thus, targeting PKCε could possibly represent an effective strategy to specifically interfere with FGFR2c oncogenic signaling, overcoming the therapeutic failures of pan-FGFR inhibitors [37]. PKCε can be easily targeted by several suppressor Micro RNAs (miRs) and inhibitory peptides, engineered these last to be specifically targeted to tumor cells of different cancers [38]. Concerning this topic, the efficacy of systemic administration of nanoparticles containing PKCε-repressive miRs in counteracting tumor growth and in conferring survival advantage have been already demonstrated in preclinical mouse models of Head and Neck Squamous Cell Carcinoma (HNSCC) [39]. Interestingly, the innovative use of nanoparticles (for the delivery of single and combinatory agents, but also for desmoplastic stroma-modulation) has been very recently proposed as a great potential strategy to overcome the limitations of current treatments towards PDAC [40]. Therefore, in future scenarios, PKCε-specific miRs and inhibitory peptides could be enrolled for innovative and more effective combined therapies in which nanoparticles will be used to specifically reach tumor cells, penetrating through the characteristic desmoplastic stroma of PDAC.

## Data Availability

All the data is provided in the article.
